# Complete genome sequences of two *Leuconostoc carnosum* strains: 4010 and AMS1

**DOI:** 10.1128/mra.00961-23

**Published:** 2024-02-05

**Authors:** Ran Li, Cédric Lood, Timo M. Takala, Gregory Andreou, Per E. J. Saris, Rob Lavigne, Xing Wan

**Affiliations:** 1 College of Food Science, Sichuan Agricultural University, Ya’an, Sichuan, China; 2 Department of Microbiology, Faculty of Agriculture and Forestry, University of Helsinki, Helsinki, Finland; 3 Laboratory of Gene Technology, Division of Animal and Human Health Engineering, Department of Biosystems, Katholieke Universiteit Leuven, Leuven, Belgium; 4 Organismal and Evolutionary Research Programme, Faculty of Biological and Environmental Sciences, University of Helsinki, Helsinki, Finland; University of Maryland School of Medicine, Baltimore, USA

**Keywords:** *Leuconostoc carnosum*, bacteriocins, genome sequences

## Abstract

*Leuconostoc carnosum* is a bacterial species commonly associated with meat spoilage. However, some strains exhibit preservative effects due to bacteriocin production. Here, we report the complete genome sequences for two strains, *L. carnosum* 4010 and AMS1. Bacteriocin-related gene clusters were found on the plasmids of both strains.

## ANNOUNCEMENT

*Leuconostoc carnosum* is a group of lactic acid bacteria often found in meat including chill-stored meats and poultry products (1–4), some strains of *L. carnosum* presented bacteriocin production properties (3, 5). Here, we report the complete genomes of two strains: *L. carnosum* 4010, isolated from vacuum-packed meat products (3); *L. carnosum* AMS1, isolated from lightly fried beef/pork minced meat in this study by soft-agar overlay method (6).

The strains were grown in calcium-citrate-sucrose medium (2% tryptone, 0.5% yeast extract, 0.4% NaCl, 0.2% sodium citrate, 0.8% calcium lactate, 0.005% MnSO4, and 1% sucrose) at 28°C. Genomic DNA (gDNA) was isolated from a 2 mL overnight culture using Qiagen MagAttract HMW DNA Kit (Qiagen, Hilden, Germany).

Isolated AMS1 gDNA was sequenced on a high-throughput sequencer Pacbio Sequel II System (Pacific Biosciences, San Francisco, CA, USA) at the DNA Sequencing and Genomics Laboratory (Helsinki Institute of Life Science). DNA template prep kit no. 2.0 and DNA/polymerase binding kit P6 (Pacific Biosciences) were used to generate 3–10 kb fragments and DNA polymerase/template library complex for sequencing. The reads were *de novo* assembled by SMRT Link 9 Analysis software (Pacific Biosciences). The PacBio pipeline generated three polished contigs with a total contig length of 1,851,151 bp (read N_50_, 16,133 bp).

The 4010 gDNA was sequenced using in-house Illumina and Nanopore technologies (KU Leuven, Belgium), and size selection was done before sequencing (7). Short reads of the genome were obtained from Illumina MiniSeq (Illumina, CA, USA) using a paired-end 2 × 150 bp approach with a DNA library prepared with Nextera Flex (Illumina). The Illumina sequencing generated 159,693 read pairs with an average length of 151  bp (approximately 48 Mbp). The Illumina reads were processed using Trimmomatic v0.39 to remove adapters and exclude sequences shorter than 50 bp (8) and the resulting data set was quality controlled using FastQC v0.11.8 (9). The same gDNA aliquot was also prepared for long reads sequencing using the Rapid barcoding kit SQK-RBK004 and sequenced on a MinION sequencer (Oxford Nanopore Technologies, United Kingdom) with R9.4.1 flow cell with Guppy (v3.1.5) as basecaller. The Nanopore reads were processed using the Nanopack software suite v1.27.0 (length >1,000 bp, phred score >10) (10), resulting in 147,216 raw reads with an average length of 9,103  bp (approximately 1,340 Mb). The 4010 genome was *de novo* assembled using the hybrid assembler Unicycler v0.4.8 (11). The obtained draft genome was polished by Pilon v1.22 (12). Default parameters were used except where otherwise indicated.

Complete genome sequences were circularized using a genome assembly program (Gap4, Staden package) (13), and overlapping ends were removed manually. Default parameters were used for all software unless otherwise noted. The annotation of the genomes was performed using the NCBI Prokaryotic Genome Annotation Pipeline (PGAP) version 4.12 (14). The detailed annotated information is listed in Table 1. The genomes were further annotated for the presence of bacteriocin-related gene clusters using BAGEL4 (15). Genes responsible for the leucocin production were found on pLC4010-2 of strain 4010 and pLCAMS1-2 of strain AMS1.

**TABLE 1:** Genome information of *L. carnosum* 4010 and AMS1^*a*^

Parameters	Data for strain:	
	AMS1	4010
BioSample accession no.	SAMN36664906	SAMN15099347
BioProject accession no.	PRJNA997118	PRJNA637255
GenBank accession no.	CP130703.1 (chromosome)	CP054418.1 (chromosome)
	CP130704.1 (plasmid pLCAMS1-1)	CP054419.1 (plasmid pLC4010-1)
	CP130705.1 (plasmid pLCAMS1-2)	CP054420.1 (plasmid pLC4010-2)
		CP054421.1 (plasmid pLC4010-3)
SRA accession no.	SRX21119575	SRX21104482
Assembly accession no.	GCA_030585795.1	GCA_030585425.1
Contig N50 (bp)	1,809,605	1,748,147
Chromosome size (bp)	1,809,605	1,748,147
Genome coverage	4,600 ×	830 ×
CDS	1,799	1,723
tRNA	68	68
rRNA	12	12
GC%	37.6	37.5
Plasmids	pLCAMS1-1: 26,558 bp, 32 CDS	pLC4010-1: 38,425 bp, 45 CDS
	pLCAMS1-2: 14,988 bp, 20 CDS	pLC4010-2: 33,269 bp, 37 CDS
	(Bacteriocin-related genes: lcnI, lcnA, lebI, lebB, lecX, lecT, lecS)	(Bacteriocin-related genes: lcnI, lcnA, lebI, lebB, lecI, lecC, lecX, lecT, lecS)
		pLC4010-3: 29,604 bp, 33 CDS

^*a*^ *lcnI*, leucocin A immunity gene; *lcnA*, leucocin A precursor gene; *lebI*, leucocin B immunity gene; *lebB*, leucocin B precursor gene; *lecI*, leucocin C immunity gene; *lecC*, leucocin C precursor gene; *lecX*, accessory gene; *lecT*, translocator gene; *lecS*, secretion gene.

## Data Availability

The complete genomic sequences are available in GenBank under the accession numbers in Table 1.
